# Supplementary material for: Saccharomyces boulardii (CNCM I-745) improves intestinal damage in sepsis by remodeling intestinal flora structure
Source: Front Cell Infect Microbiol. 2025 Jul 31;15:1602792. doi: 10.3389/fcimb.2025.1602792 (PMC12350329; doi:10.3389/fcimb.2025.1602792)
Supplement: Supplementary file 1 [file DataSheet1.zip › summary/summary/8_Advanced_analyse/2_PICRUSt2_result/KEGG/Group.AW4_vs_AW0/PICRUSt2_KEGG_level3_Group.AW4_vs_AW0_diff.pdf]

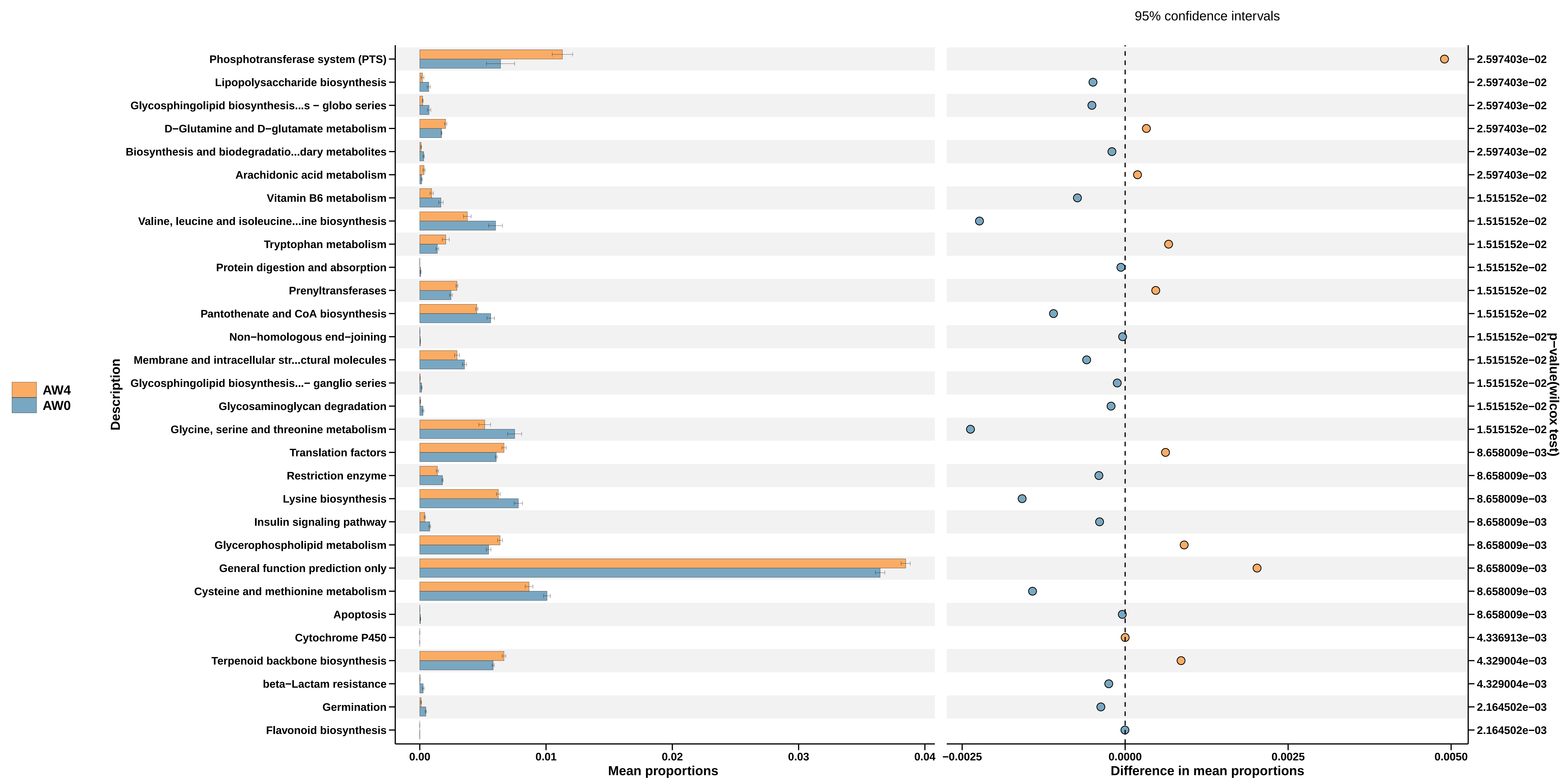
95% confidence intervals
AW4
AW0
Description
Phosphotransferase system (PTS)
Lipopolysaccharide biosynthesis
Glycosphingolipid biosynthesis...s − globo series
D−Glutamine and D−glutamate metabolism
Biosynthesis and biodegradatio...dary metabolites
Arachidonic acid metabolism
Vitamin B6 metabolism
Valine, leucine and isoleucine...ine biosynthesis
Tryptophan metabolism
Protein digestion and absorption
Prenyltransferases
Pantothenate and CoA biosynthesis
Non−homologous end−joining
Membrane and intracellular str...ctural molecules
Glycosphingolipid biosynthesis...− ganglio series
Glycosaminoglycan degradation
Glycine, serine and threonine metabolism
Translation factors
Restriction enzyme
Lysine biosynthesis
Insulin signaling pathway
Glycerophospholipid metabolism
General function prediction only
Cysteine and methionine metabolism
Apoptosis
Cytochrome P450
Terpenoid backbone biosynthesis
beta−Lactam resistance
Germination
Flavonoid biosynthesis
0.00
0.01
0.02
0.03
0.04
Mean proportions
−0.0025
0.0000
0.0025
0.0050
Difference in mean proportions
p−value(wilcox test)
2.597403e−02
2.597403e−02
2.597403e−02
2.597403e−02
2.597403e−02
2.597403e−02
1.515152e−02
1.515152e−02
1.515152e−02
1.515152e−02
1.515152e−02
1.515152e−02
1.515152e−02
1.515152e−02
1.515152e−02
1.515152e−02
1.515152e−02
8.658009e−03
8.658009e−03
8.658009e−03
8.658009e−03
8.658009e−03
8.658009e−03
8.658009e−03
8.658009e−03
4.336913e−03
4.329004e−03
4.329004e−03
2.164502e−03
2.164502e−03
